# A Systematic Review of Healthy Nutrition Intervention Programs in Kindergarten and Primary Education

**DOI:** 10.3390/nu15030541

**Published:** 2023-01-20

**Authors:** Rocio Collado-Soler, Marina Alférez-Pastor, Francisco L. Torres, Rubén Trigueros, Jose M. Aguilar-Parra, Noelia Navarro

**Affiliations:** 1 Hum-878 Research Team, Health Research Centre, Department of Psychology, University of Almería, 04120 Almería, Spain; 2 Department of Language and Education, University of Antonio de Nebrija, 28015 Madrid, Spain

**Keywords:** obesity, overweight, diet, healthy eating, nutrition, behavior, intervention program

## Abstract

Childhood obesity and overweight rates are increasing in an exponential way. This type of diet-related health problem has consequences, not only at present but also for children’s future lives. For these reasons, it is very important to find a solution, which could be nutrition intervention programs. The main objective of this article is to investigate the effectiveness of nutrition intervention programs in children aged 3–12 around the world. We used SCOPUS, Web of Science, and PubMed databases to carry out this systematic review and we followed the PRISMA statement. Two authors conducted literature searches independently, finding a total of 138 articles. Finally, after a thorough screening, a total of 19 articles were selected for detailed analysis. The results show that, in general, nutrition intervention programs are effective in improving knowledge and behaviors about healthy habits, and, consequently, that the body mass index value is reduced. However, it is true that we found differences between the incomes of families and geographical areas. In conclusion, we encourage school centers to consider including these types of programs in their educational program and bring awareness of the importance of families too.

## 1. Introduction

One of the main educational objectives is to promote people healthy lifestyle habits among young related to the Mediterranean diet and the regular practice of physical activity. However, diet-related health problems have a high incidence in children and adults [1,2]. Diet is an important risk factor that can be modified, having a significant impact on people’s health [3]. In fact, eating behavior (such as food choices, and eating events and habits) establishes the health status of people [4]. The most common of these problems are overweight and obesity, which have increased in children in the last few years. This has occurred not only in the richest countries but also in poorer countries [5,6], and the incidence continues to grow. Since 1975, universal obesity has almost tripled [1]. In 2020, 340 million children aged 5–19 and 38.9 million children aged under 5 were obese or overweight [1]. 

An imbalance between energy intake and expenditure causes overweight and obesity. Energy expenditure cannot be exceeded by energy intake. In consequence, diet quality is essential to maintaining body mass index (BMI) [7]. 

Nevertheless, there are other diet-related health problems, such as stunting or wasting. A child is stunted when they are too short for their age; a child is wasted when they are too thin for their height (underweight). Even though the prevalence of stunting in children aged under 5 has decreased from 33.1% in 2000 to 22% in 2020, 149.2 million children suffered it in 2020 and 45.4 million suffered from wasting [8].

### 1.1. Influence of the Socialization

The responsibility for these data is with the eating habits, including food and physical activity [9]. However, they are influenced by the food industry, which advertises unhealthy food and beverage [10] through the media. The nutritional transition process, which is determined by social changes like industrialization, has changed. Years ago, diets were based on fruit, vegetables, legumes, and grains; nowadays, ultra-processed foods are eaten by everyone.

At the present, the food preferences of children are changing, influenced by the exponential development of information and communication technologies (ICT). Advertisements in any media (TV, social media, mobile phones…) and food delivery online platforms promote junk food that contains high salt, sugar, and fat contents [11]. In fact, the most consumed beverages and foods by children have high sugar content. The problem is that diet-related health problems are associated with excessive sugar, salt, and fat consumption [12]. In addition, children spend their time using electronic devices instead of doing physical activities [13]. These data ratify that level of physical activity practice is below the World Health Organization’s (WHO) recommendation [14]. Thus, it is evident that ICT does not help to maintain a healthy diet.

Nevertheless, there are other influences such as friends, teachers, and parents’ attitudes toward food [15]. Independently of the setting, it is very important for children to have good health habits to promote good health habits in adulthood [16].

### 1.2. Consequences of Diet-Related Health Problems

Eating healthy foods and practicing physical activity reduce the risk of diet-related health problems [17]. Additionally, they could decrease anxiety and depression, and improve academic performance, socialization, or self-esteem [18,19]. The benefits of good nutrition are numerous, but the disadvantages of eating an unhealthy diet are also important to know. Obesity during childhood has consequences, both immediately and in the long-term. It can provoke hypertension, diabetes, and cardiovascular diseases later in life [20], and contributes to later adulthood obesity too [21].

Annually, 17.9 million people die because of cardiovascular diseases [22] which could be provoked by poor diet, over-nutrition, high blood sugar and lipids, low physical activity, or smoking, among other activities [23]. A reduction of these risk factors in daily life could avoid premature deaths [24].

These are the main problems that society faces, and that is why a feasible solution is necessary. Health intervention programs at schools could be a good answer. Childhood is the best period to teach good nutrition because of rapid mental and physical growth.

### 1.3. Healthy Nutrition Intervention Programs at Schools

Since youngsters spend most of their time at school and their behaviors are influenced by the school environments, educational centers are the best setting in which to carry out this type of intervention [25,26].

School food programs should contribute to child health and well-being [27] and to the promotion of healthy eating patterns [28]. They empower students with the knowledge and attitudes necessary to maintain healthy nutrition in their lives [29]. Although it is true that some countries do not include nutrition in their curriculum (such as Indonesia), these programs could take place in every single school. 

These programs should be carried out as early as possible because adult eating habits are developed from an early age [30,31]. The World Health Organization has affirmed that they are efficient in changing eating habits [32] in 1997, but we want to verify if they continue to be effective.

### 1.4. Objetive

In spite of the educational programs related to balanced nutrition, it is important to analyze the impact it is having on young people between the ages of 3 and 12. During this period, habits take place that will have a significant impact on adolescence. Therefore, the main objective of this systematic review is to investigate the effectiveness of nutrition intervention programs in children aged 3–12 years worldwide. 

## 2. Materials and Methods

The statement of the Preferred Reporting Items for Systematic Review and Meta-Analyses 2020 (PRISMA statement) [33] was considered in the process of searching and writing this article.

### 2.1. Literature Search

After the agreement about the string search, two authors started the literature search independently. The databases SCOPUS, Web of Science, and PubMed were screened from September to December 2022 to compile the most complete list of studies possible. We looked for relevant articles about nutrition education programs in pre-primary and primary schools published from January 2013 to now. We use two clear and careful descriptors strings, one for each educational stage:

- (“PRE” AND “POST” OR “intervention program”) AND (“dietary behaviour” OR “healthy food” OR “healthy eating” OR “healthy diet” OR “healthy nutrition” OR “healthy habits”) AND (“children” “education” OR “early childhood education” OR “childhood education” OR “childhood school” OR “pre-primary education” OR “infant education” OR “infant school” OR “kindergarten education” OR “nursery education” OR “preschool education” OR “early education”);
- (“PRE” AND “POST”) AND (“dietary behavior” OR “healthy food” OR “healthy eating” OR “healthy diet” OR “healthy nutrition” OR “healthy habits”) AND (“primary school” OR “elementary school” OR “primary education” OR “elementary education” OR “primary students” OR “elementary students”).

In the primary string search, we found 2 articles about pre-primary education.

### 2.2. Inclusion and Exclusion Criteria

The main criteria for this review were nutrition intervention programs in pre-primary and primary schools over the world. During the selection process the following criteria has been considered:

- Articles from scientific journals were accepted, whereas other types of studies such as gray literature were refused;
- Participants sample had to be made up of preschool of school children, and studies with post-primary, teachers or families as samples were refused;
- Experimental or quasi-experimental articles were accepted, while articles without an intervention program, a pre- and post-intervention, or experimental group were refused;
- Nutrition intervention programs in pre-primary or primary schools were included, whereas we refused articles whose variables were different from eating diet, for instance, physical activity, food wasted, smart devices, hand washing, academic achievement, or teachers’ attitudes, among others;
- Articles with open access and the Spanish or English language were included. We refused articles to which we did not have full access or which were written in other languages.

### 2.3. Article Selection and Data Extraction

**Figure 1:**
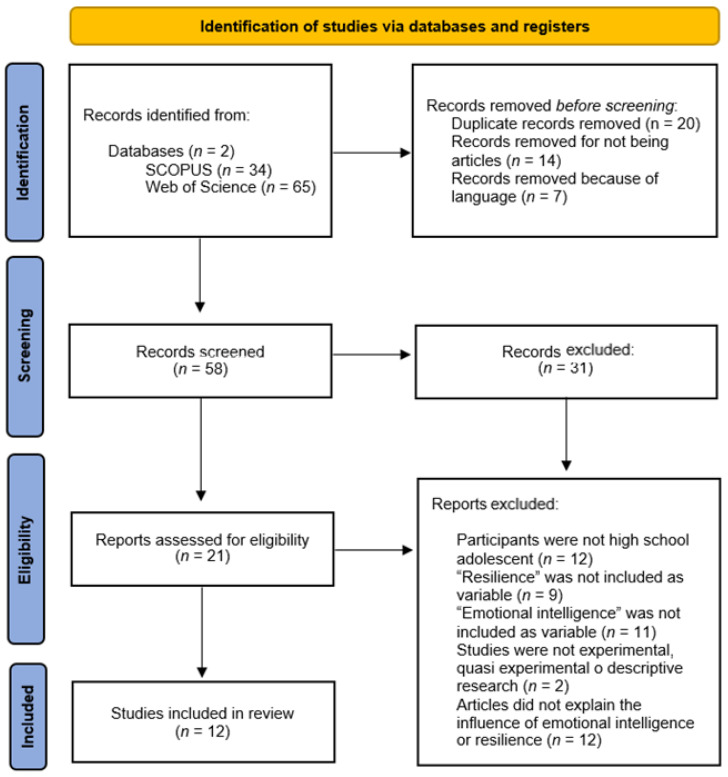
Flow diagram of the articles during the selection process.

Firstly, we found 17 articles for pre-primary education and 121 for primary education. Removing articles published before 2013, studies repeated in two or more databases, and gray literature, we screened 74 articles in total. Later, we applied the inclusion criteria mentioned before and 42 studies were selected for a full reading. Finally, 19 articles published in scientific journals were included in our systematic review. This process is summarized in Figure 1. 

The same two authors extracted the following data from the 19 articles selected: authors’ names, year of publication, instruments used, variables measured, sample, country, and main findings. Then, a third author validated the information.

### 2.4. Study Quality Assessment

The literature search and article selection were carried out by two authors independently. Some disagreements were found, but they were solved by the consensus of the two authors. We divided the number of agreements by the total number of agreements plus disagreements, then we multiplied the answer by 100 to obtain the inter-rater reliability percent, which resulted in 95%.

## 3. Results

### 3.1. Study Characteristics

From the 19 articles that were selected for our systematic review, some data were extracted to know the characteristics of the studies (Table 1). In this sense, we can say that there is a great variety across geographic location. A total of 5 articles were from the United States [34,35,36,37,38], 2 from Malaysia [39,40], 2 from China [41,42], while the rest of countries did not appear in more than one study. The most studied continents were North America and Asia, with 6 out of 19 each continent, closely followed by Europe (5 articles). Most of the American articles selected were developed in the United States (5 out of 6).

The age range was from 4 to 14 years old. However, most of the studies had a population aged from 6 to 12 years old, as they focused on primary education. Focusing on this stage, children aged 9 years old were the most represented group of the studies analyzed (in 73.68% of the articles). Regarding the instrument, children’s and parents’ questionnaires and surveys were the most used instruments [34,36,37,38,39,40,42,43,44,45,46,47,48,49,50].

### 3.2. Outcomes

The effectiveness of a nutrition program depends on its aims, activities, methods, and program content, among other factors [53]. That is why we found a great variety of intervention programs. According to the articles selected, we can group nutritional programs into three categories: (1) education component programs (e.g., classroom-based learning, educational modules, and supportive educational materials), (2) practical component programs (e.g., the availability of fruit and vegetable, participation in nutritional campaigns and games), and (3) educational and practical component programs (e.g., theoretical lessons through video lessons or video laboratories, implementation of contents through meaningful activities such as gamified challenges or cooking workshops). This review included 19 intervention studies that reported a significant increase in children’s consumption of a healthy diet, mainly consisting of fruit and vegetables. Overall, 6 studies of these articles included only educational components [35,36,40,41,47,51], 6 studies used practical approaches [37,38,42,44,48,50], and 7 included both approaches [34,39,43,45,46,49,52]. The intervention programs and their duration can be seen in Table 2.

#### 3.2.1. Nutritional Knowledge

In Table 3, we observe that nutrition intervention programs increase children’s nutritional knowledge [40,49], especially about the appropriate daily frequency of fruit and vegetable consumption [46], and the sugar content of certain foods [35]. In addition, this knowledge about healthy habits increases, especially among children living in low-income neighborhoods [47]. On the contrary, Sharma et al. found that the EG decreased their nutrition and physical activity knowledge [37]. Finally, these school nutrition practices increase students’ knowledge of active lifestyles and healthy diets [50]. This knowledge increases due to the theoretical lessons and practical activities on the subject of nutrition that take place in class [40,42]. 

#### 3.2.2. Body Weight and Body Mass Index (BMI)

In Table 4, we find that children’s participation in nutrition intervention programs causes significant changes in anthropometric variables such as weight, in some cases, over time [40]. The prevalence of normal weight increased, while the prevalence of overweight, obesity, or thinness decreased after the implementation of these programs [40,50]. 

In addition, overweight children who participated in these programs reduced their BMI score [39,46]. Additionally, children who were in schools that followed a free fruit and vegetable policy had a better BMI [44]. On the other hand, boys of parents with no higher education had an elevated BMI and a higher chance of being overweight or obese, while girls of parents with no higher education had a lower BMI and a lower chance of being overweight or obese [44]. In turn, Nickel et al. affirmed that boys that lived in rural areas experienced a greater reduction in waist circumference (WC) [47]. 

Finally, these programs make children do physical activity more frequently, so they can prevent and/or treat overweight and obesity in this population [37,39,46]. However, Sharma et al. did not find significant effects of a video game-based nutritional program on physical activity [37]. 

#### 3.2.3. Eating Behaviors

In Table 5, we observe that the nutritional quotient (NQ) score increased significantly after participating in these nutrition intervention programs, in relation to diet quality, dietary attitudes, and healthy eating behaviors [42,42,51]. Moreover, we find behavioral improvement with respect to the daily frequency of fruit and vegetable consumption [45,46,51] in both children [38] and parents [34], moderation in the intake of hypercaloric foods [51], changes in the regularity of meals away from home [51] and the frequency of eating five meals per day [39], and adherence to the Mediterranean diet [43]. Verdonschot et al. disagreed, and they did not find significant differences in fruit and vegetable consumption between EG and CG [49]. 

On the other hand, participation in these programs increased self-confidence in cooking, enjoyment of food and cooking, frequency of cooking at home, availability of fruits and vegetables at home, use of nutrition labels when shopping, and knowledge of bedtime routines [34,35]. Moreover, food waste [45], total fat intake [34], and the percentage of daily calories from morning and afternoon snacks [52], such as cookies, cakes, chocolates [37,48], and sugary drinks [34], were reduced. 

Finally, both children living in urban areas and those living in higher-income neighborhoods improved dietary intake self-efficacy [38,47], which may improve children’s cognitive performance [39]. However, Siew et al. and Lin et al. did not find significant changes in children’s nutritional attitudes [40,50].

## 4. Discussion

This systematic review has given an analysis of the effects of nutrition education programs on children’s nutrition knowledge and eating behaviors. Through this research, we found several types of programs about nutrition at schools which the latter used to obtain different dietary outcomes. Regarding the literature, nutrition education programs increase knowledge and food attitudes [54], practical activities during the programs encourage an increase in physical activities [55] or healthy eating behaviors. However, the most effective programs are those that join both education and practice [56]. These findings are in line with most of the articles selected [34,35,38,39,43,44,45,46,47,48,49]. However, we also found some educational programs which improved the practice scores [40] or eating behaviors [41,51] and practical programs that fostered knowledge [37,42,50].

It is very important that FV policies are promoted in educational environments. In fact, exposure to FV has been shown to contribute to effective nutrition education programs [57]. We found that children which were exposed to FV provision [44,49] or FV education [38,45,49] improved their BMI, FV intake, nutrition knowledge, and behaviors. Previous studies of free fruit and vegetables (FFV) showed a reduction in BMI mean score, a reduction in school-level obesity [58], and an increase in FV intake [59,60,61]. The interventions selected are heterogeneous among the results of prior meta-analyses and systematic reviews [62,63]; however, all of them have adequate alignment among intervention, objectives, and findings, which is an important aspect to be effective [57].

In this sense, the combination of FV provision and education [49] seems to be more effective in eating habits, indeed earlier studies affirmed that education improves nutrition knowledge [64,65]. On their part, Franceschi et al. used an app to prevent overweight and obesity, resulting in an increase in nutritional knowledge [46]. The use of technology for these intervention programs showed that they are beneficial in short-term weight loss [66,67]. However, other non-technological programs also had good results. We found that children’s knowledge improved and consequently they reduced food waste [45]. The knowledge improvement could be seen in nutrition [35,37,40,42,46,49,50], bedtime routine knowledge [35], physical activity knowledge [37], and healthy living in general [47,50]. This is consistent with previous research which focused on the knowledge about healthy eating habits [68,69]. Nutrition knowledge is very important in all stages of our lives because it fosters the awareness of eating and, therefore, inspires people to practice healthy eating [70,71].

Regarding eating behaviors and physical activity, we find some articles that reported changes in behavior [34,37,38,39,41,42,43,45,46,47,48,51], as Weber et al. [72] affirmed in Germany. Regular meal intake, especially at breakfast time, is associated with better overall food consumption [73,74], and it is essential to improving eating behavior [75,76] and physical activity [77]. This regular meal intake was significantly greater in EG [51], so we can affirm that intervention programs help to improve food intake. In this sense, the results of Teo et al. show that, after an intervention program, the frequency of healthy breakfasts, lunches, and dinners increases, improving physical activity frequency and decreasing BMI scores [39]. Moreover, the consumption of cookies, cakes, and chocolates decreased significantly [48]. In general, unhealthy snacks were less consumed after nutrition intervention programs [34,37,48,51] as was confirmed previously [78].

It was believed that nutritional knowledge fosters healthy behaviors [79]. However, in Siew et al. [40] and Lin et al. [50], we do not find significant differences in attitudes while knowledge improves [40]. On the contrary, we found significant behavior, attitudes, and knowledge changes in other articles [42,51]. 

Nevertheless, depending on the socio-economic groups and geographical regions there are differences in eating behaviors and, consequently in obesity rates [80]. Our results are in line with that statement. Indeed, Nickel et al. affirmed that children in low-income neighborhoods only showed improved nutrition knowledge, while children in higher-income neighborhoods improved their consumption [47]; Øvrebø et al. found differences between parents’ education, resulting in different BMI [44]; and Qian et al. confirmed differences between provinces, whereby there was a better improvement in eating behaviors in higher-income families [41].

All the variables mentioned influence the body mass index (BMI), which allows us to know if a person is underweight or overweight. Through the articles selected, we find changes in BMI depending on different programs [36,40,44,46,51]. Consistent with the other two intervention programs, the BMI is lower in EG than in CG after a nutrition intervention [81,82]. In a literature search, we found contrary opinions about this topic, with some articles emphasizing the effectiveness of these types of programs [83,84] while others affirmed that these interventions were not effective [85,86]. In this sense, our results show that in two intervention programs, there were no significant differences [36,40] while three of them had significant changes and differences. This is corroborated by a previous study, which showed that a 5-2-1-0 intervention program [87] decreases the BMI percentile. A higher BMI is associated with obesity and overweight, and this is the main reason to consider implementing nutrition intervention programs at schools [88].

In short, nutrition intervention programs at schools have a strong impact on children’s eating. The main objective of this study was to check the effectiveness of these programs in impacting children’s nutrition knowledge and behaviors. As we verified, they improve students’ knowledge about healthy diets and foster healthy attitudes and behaviors in their lives. School environments and families are essential in promoting healthy habits and modifying dietary behaviors [89,90]. However, it is more important to include fun and interactive activity-based nutrition sessions [91] because attitude, which is influenced by motivation [92], mediated eating behaviors [93].

## 5. Conclusions

Despite the results achieved in our systematic review, the impact is rather limited. In this sense, we included articles from all continents except Antarctica, but they are not representative of global nutrition education programs because there is only one article from Oceania and one from Africa. On the contrary, we found a wide variety of articles from countries in Europe and Asia, but not in America. 

This is due to the limitations of year of publication and type of publication that reduce the number of possible studies, but the aim was to make the selected articles as relevant and current as possible. In fact, this was the usual reason for eliminating articles not published in peer-reviewed journals and articles published before 2013. On the other hand, we followed the PRISMA methodology, which is a great method in social science research because of its transparency and openness to suggestions and comments.

This systematic review could have important educational implications. In this sense, the promotion of healthy lifestyle habits related to healthy and balanced eating is an educational objective. Therefore, future work could analyze the influence of different types of methodologies that help to reinforce behaviors related to the consumption of healthy food. 

## Figures and Tables

**Table 1:** Characteristics of the studies selected.

Authors	Sample	Country	Variables	Instruments
García and Fernández 2022 [43]	38 students 6–7 years old	Spain	- Gamification experience and gender - Adherence to the Mediterranean diet	- Mediterranean diet adherence questionnaire to analyze participants’ nutritional variables
Øvrebø et al. 2022 [44]	11215 students 8–13 years old	Norway	- Age - Weight - Height - Waist circumference (WC) - Parents’ education level	- Tape measure - Electronic fat control scale - Wall-mounted stadiometer with 0.1 cm accuracy for height measurement - Questionnaire for parents on level of education
Karpouzis et al. 2021 [45]	20 students 10–12 years old	Australia	- Dietary patterns - Nutrition knowledge - Cooking skills - Food-wasting behaviors	- Surveys and questionnaires on child nutrition, cooking skills, and food waste behavior
Franceschi et al. 2021 [46]	695 students 6–14 years old	Italy	- Weight - Height - BMI - Socio-economic status - Physical activity level - Nutritional habits	- Questionnaires on knowledge about nutrition and physical activity - Questionnaires on children’s eating habits
Teo et al. 2021 [39]	523 students 7–11 years old	Malaysia	- Socio-demographic data - Dietary behavior - Physical activity level - Weight - Height - Cognitive performance	- Questionnaires for parents on the demographic characteristics of their children - Eating Behavior Questionnaire (EBQ) - Physical Activity Questionnaire for Children (PAQ-C) - Seca scale to measure body weight - Seca stadiometer to measure the height - Raven’s Colored Progressive Matrices (CPM)
Nickel et al. 2021 [47]	311 students 6–12 years old	Canada	- Height - Weight - BMI - Sex - Family income level - Urban/rural residence - Dietary intake - Knowledge of healthy living - Level of self-efficacy	- Flexible measuring tape - Portable floor scale - Stadiometer - 26 questions on knowledge of healthy or unhealthy behaviors - 2-point scale to report how often they eat certain foods and to evaluate their level of self-efficacy
Ben Cheikh et al. 2020 [48]	558 students 6–12 years old	Tunisia	- Calories consumed in the morning snack	- Questionnaire for parents on demographic characteristics and eating habits of their children - Questionnaire for children on snacks consumed and their composition
Marshall et al. 2020 [34]	717 students 5–7 years old	United States	- Socio-demographic data - Dietary intake	- Food Frequency Questionnaire - Block Kids Food Screener (BKFS) - Surveys for parents to collect information on fruit and vegetable intake and the home nutrition environment
Verdonschot et al. 2020 [49]	1237 students 7–12 years old	Netherlands	- Nutrition knowledge - Fruit and vegetable intake - Personal characteristics - School characteristics - School Food Policy	- 24 questions about nutrition knowledge - 24 h recall suggested by Haraldsdóttir et al. (2005) - Child questionnaire about age, sex, and grade - Questionnaire for teachers about school characteristics
Siew et al. 2020 [40]	152 students 9 years old	Malaysia	- Nutritional issues and concerns - Body weight and height - Socio-demographic data	- Immediate Impact Survey (IIS) - Nutrition Knowledge, Attitude, and Practices survey (KAP) - Nutritional status assessment
Fisher et al. 2019 [35]	57 students 5–6 years old	United States	- Children’s nutrition knowledge	- Oral questions and direct observation
Lin et al. 2019 [50]	201 students 8–12 years old	Taiwan	- Interest and satisfaction with the program - Nutrition Knowledge - Healthy Eating Active Living (HEAL) - Children’s height and weight	- MX’s routine questionnaire - 5-point Likert scale about satisfaction - Six multiple-choice questions for physical activity knowledge - Eight multiple-choice questions on healthy diet knowledge
Qian et al. 2019 [41]	4482 students 8–9 years old	China	- Eating behaviors	- Nine outcome measures based on The Guidelines for Nutrition and Diet for Chinese Residents
Kim et al. 2019 [51]	524 students 4–5 years old	South Korea	- Health - Weight - BMI - Eating behaviors	- Height was measured to the nearest tenth of a centimeter (cm) with a mobile - The weight was measured to the nearest tenth of a kilogram (kg) on a portable digital scale - BMI was calculated with the standard equation (body weight [kg]/height [m]^2^) - Children’s eating behavior was assessed by nutritional quotient (NQ)
Brown et al. 2018 [52]	404 students 6–11 years old	Scotland	- Health behaviors	- Height and weight test based on Child Measurement Program Operational Guidance - Questionnaire about health attitudes and behaviors
Zhou, 2018 [42]	65 students 10–12 years old	China	- Age - Mother’s and father’s jobs and education - Nutritional knowledge - Nutritional attitude - Nutritional behavior	- Demographic questionnaire - Nutrition Behavior Inventory
Lynch et al. 2016 [36]	51 students 7–9 years old	United States	- Health habits - Number of steps	- Healthy Habits Survey - Pedometer - Demographic Survey
Sharma et al. 2015 [37]	94 students 8–12 years old	United States	- Dietary intake - Nutrition habits - Physical Activity habits - Attitudes toward computer-assisted instruction - Height and weight	- Two random 24 h dietary recalls using the validated Coordinated Approach to Child Health (CATCH) - Child self-report surveys about nutrition and physical activity habits - Attitude Toward Computer-Assisted Learning Scale - Standard protocols to measure child height and weight
Song et al. 2015 [38]	665 students 8–11 years old	United States	- Self-reported FV intake - The perceptions of food service staff about what children eat	- Student preference and consumption survey - Food service staff survey

BMI: Body Mass Index; FV: Fruits and Vegetables.

**Table 2:** Characteristics of the intervention programs.

Authors	Description Intervention	Duration of Sessions
García and Fernández 2022 [43]	Effectiveness of the gamified proposal “Save the Mediterranean Diet”. EG. The contents are presented to the students through audiovisual support. Then, they have to overcome the gamified mission. CG. Nothing was provided.	5 sessions (1 per week in consecutive weeks).
Øvrebø et al. 2022 [44]	Effectiveness of a program based on free fruit and vegetable (FFV) school policies. EG. Provide a daily serving of free fruit or vegetable at lunchtime. CG. Nothing was provided.	One piece of FV per day for 4 years (follow-up during the years 2010, 2012, 2015, and 2017).
Karpouzis et al. 2021 [45]	Effectiveness of the Food Education and Sustainability Training (FEAST) program. EG. Inquiry learning and role model concepts are used to incorporate classroom cooking activities into the English and STEM curriculum. CG. Nothing was provided.	10 sessions in consecutive weeks (1 session per week, 1.5 h per lesson).
Franceschi et al. 2021 [46]	Effectiveness of the project “Smuovi La Salute”. EG. Activities that help to reduce cultural and socio-economic inequalities. The use of an app, a cookbook with healthy multicultural recipes, nutritional video lessons, and video laboratories, and physical activities in urban settings. CG. Video viewing.	30 months (Phase 1, 19 months; phase 2, 11 months).
Teo et al. 2021 [39]	Effectiveness of the School Nutrition Program (SNP) through the presentation of 17 topics, which included 4 aspects worked on through nutrition practices: health, nutrition, physical activity, and food hygiene. EG. Participation in school nutrition campaigns and healthy menu serving during school breaks. CG. Nothing was provided.	6 h monthly campaigns every 3 months and a menu with cereals, vegetables, fruits, and protein foods every day for 3 months.
Nickel et al. 2021 [47]	Effectiveness of the Healthy Buddies intervention. EG. 21 healthy living lessons. CG. Standard health curriculum.	21 lessons in consecutive weeks.
Ben Cheikh et al. 2020 [48]	Effectiveness of a nutritional intervention program. EG. Several workshops (tasting, labeling, cooking…). Open day for children and parents with workshops to choose the best healthy and balanced dish. Drawing, writing, and poetry contests on healthy nutrition. SMS to parents focuses on the importance of promoting healthy eating. Facebook group to disseminate educational messages on healthy eating. CG. Nothing was provided.	5 months.
Marshall et al. 2020 [34]	Effectiveness of the Brighter Bites program. EG. Nutrition education lessons and weekly recipe tastings. CG. Nothing was provided.	16 weeks during a school year.
Verdonschot et al. 2020 [49]	Effectiveness of two different nutrition education programs. EG1. FV provision and education. EG2. FV provision. CG. Nothing was provided.	EG1: five lessons (including experiments, cooking, and tasting). EG2: three pieces of fruit per week for 20 weeks.
Siew et al. 2020 [40]	Effectiveness of a nutrition educational package. EG. HKP educational package with three educational modules (including health awareness, nutrition, physical activity, and hygiene), and supportive educational materials (such as games, PowerPoint presentations, goal cards…). CG. Nothing was provided.	Six times a year for 3 years (each session lasts one hour).
Fisher et al. 2019 [35]	Effectiveness of Sprouts’ curriculum. EG. Activities and book-reading about energy balance, healthy sleep habits, and food groups. CG. Nothing was provided.	8 lessons.
Lin et al. 2019 [50]	Effectiveness of “Train Like an Astronaut Program” EG. 8 challenges about training students with exercises, improving their knowledge, and completing circuit training. CG. Nothing was provided.	8 sessions in consecutive weeks (40 min per session).
Qian et al. 2019 [41]	Effectiveness of a comprehensive nutrition education program. EG. Nutrition lessons and activities integrated into students. CG. Nothing was provided.	For 1 school year with flexibility in the duration and number of sessions to accommodate local conditions.
Kim et al. 2019 [51]	Effectiveness of the Mission X program (train like an astronaut). EG. Activities and materials (including food-balanced meals, observation methods, educational movie clips, and healthy/unhealthy snacks) on four thematic nutrition components: energy on an astronaut, hydration station, living bones, and reduced gravity. CG. Key concepts of the nutrition sessions.	4 sessions for 10 weeks.
Brown et al. 2018 [52]	Effectiveness of 10-week lifestyle intervention. EG. 45 min aligned with the Scottish Curriculum and 45 min consisted of fun games, exercises, and sports. CG. Nothing was provided.	10 sessions in consecutive weeks (90 min per session).
Zhou, 2018 [42]	Effectiveness of nutritional behaviors intervention program. EG. Videos, and role play, among other activities about healthy and unhealthy food. CG. Nothing was provided.	4 sessions on consecutive days (45 min per session).
Lynch et al. 2016 [36]	Effectiveness of 5-2-1-0 Educational curriculum. EG. The standardized 5-2-1-0 curriculum was taught. CG. Nothing was provided.	8 sessions for a 4-month period.
Sharma et al. 2015 [37]	Effectiveness of the Quest to Lava Mountain (QTLM). EG. They used an immersive three-dimensional action-adventure game CG. Nothing was provided.	90 min per week for 6 weeks.
Song et al. 2015 [38]	Effectiveness of a multilevel intervention EG1. Comprehensive group. They received the ReFresh program (nutrition education and behavioral economics-based cafeteria changes). EG2. The cafeteria group. They only received behavioral economics-based cafeteria changes. CG. Nothing was provided.	8 classroom-based nutrition education units (4 lessons per unit) for 1 scholar year.

EG: Experimental Group; CG: Control Group; FV: Fruits and vegetables; STEM: Science, Technology, Engineering, and Mathematics; SMS: Short Message Service; HKP: Healthy Kids Program.

**Table 3:** Results about nutritional knowledge.

Authors	Results
Franceschi et al. 2021 [46]	- There was a significant increase of 35% (from 69.3 to 93.5%) in nutritional knowledge about the correct daily frequency of vegetable and fruit consumption.
Nickel et al. 2021 [47]	- EG participants who were boys had improved knowledge of healthy living (5.9; 95% CI [2.3, 9.5]). - EG participants who lived in low-income neighborhoods had improved knowledge of healthy living.
Verdonschot et al. 2020 [49]	- There was a significant increase in EG1 children’s nutrition knowledge (*p* < 0.01) and there was a significant difference with CG (*p* < 0.05). In EG2 the differences were not significative.
Siew et al. 2020 [40]	- In the mean knowledge score there was a significant increment in EG (*p* < 0.01) and there was a significant difference between groups (*p* < 0.05) over the 3 years in favor of EG.
Fisher et al. 2019 [35]	- In EG, there were significant differences between pre- and post-test in farm-to-table knowledge (*p* < 0.001), and sugar content of beverages knowledge (*p* = 0.01).
Lin et al. 2019 [50]	- The nutrition knowledge increased in both groups. However, the differences between groups are significant in healthy diet knowledge (*p* = 0.044) and in active lifestyle knowledge (*p* = 0.002), in favor of EG.
Zhou, 2018 [42]	- In EG there was a significant increase in nutritional knowledge (*p* = 0.04).
Sharma et al. 2015 [37]	- The EG showed a decrease in nutrition/physical activity knowledge with significant differences in comparison to CG (*p* = 0.0338).

EG: Experimental Group; CG: Control Group; CI: Confidence Interval.

**Table 4:** Results related to body weight and BMI.

Authors	Results
Øvrebø et al. 2022 [44]	- Boys exposed to the FFV policy (EG) had a 0.05 higher BMI (95%CI: −0.04, 0.14). - Girls exposed to the FFV policy (EG) had a 0.04 higher BMI (95% CI: −0.04, 0.13). - Boys of parents without higher education had elevated BMI (+0.12, *p* = 0.004), increased odds ratio (OR) of overweight/obesity (OR 1.66, *p* = 0.002), and higher overweight (+0.7 cm, *p* = 0.05). - Girls of parents without higher education had lower BMI (−0.20; 95% CI: −0.41, 0.01) and lower odds of overweight/obesity (OR 0.55; 95% CI: 0.27, 1.12) if they had attended a school with this program (*p* = 0.05).
Franceschi et al. 2021 [46]	- There was an increase in physical activity by 63% after the project (from 54.5 to 88.9%, *p* < 0.001) in EG. - Overweight children and adolescents reduced their BMI score by −0.17 ± 0.63.
Nickel et al. 2021 [47]	- EG participants who were low-income, boys, or living in rural areas experienced a greater reduction in WC (−1.7 cm; 95% CI [−2.8, −0.5 cm])
Teo et al. 2021 [39]	- The EG showed more frequent physical activity compared to the CG (*p* < 0.05). - At a 3-month follow-up, the EG showed lower BMI scores (*p* < 0.05).
Siew et al. 2020 [40]	-Significant changes in weight and BMI over time occurred in each group (*p* < 0.05): The prevalence of normal weight increased in the EG at the post-test, but not in the CG. The prevalence of overweight decreased in the EG at the post-test, but not in the CG. The prevalence of thinness at post-test decreased in both groups, but in the EG the decrease was greater. The CG had a higher prevalence of obesity and overweight than the EG at the post-test.
Kim et al. 2019 [51]	- There were significant changes in anthropometric variables in the EG (*p* < 0.05).
Lin et al. 2019 [50]	- The prevalence of overweight and obesity decreased in EG at post-test, but not in CG.
Sharma et al. 2015 [37]	- Physical activity attitude had significant differences between groups (*p* = 0.041), and it increased from pre- to postintervention. - There were no significant effects of Quest to Lava Mountain (QTLM) on physical activity. CG and EG improved their results but there were no significant differences between the groups.

FFV: Free Fruits and Vegetables; BMI: Body Mass Index; EG: Experimental Group; CG: Control Group; CI: Confidence Interval; OR: Odds Ratio; CM: Centimeters; WC: Waist Circumference.

**Table 5:** Results related to eating behaviors.

Authors	Results
García and Fernández, 2022 [43]	- Adherence to the Mediterranean diet was slightly higher in the EG (pre-test 6.7 and post-test 7.8) than in the CG (pre-test 8.1. and post-test 8.1). - Adherence to the Mediterranean diet is slightly higher in the group of girls (pre-test 7.8 and post-test 8.4) compared to the group of boys (pretest 6.9 and posttest 7.6).
Karpouzis et al. 2021 [45]	- In EG there was an increase in self-confidence in cooking, the pleasure in food and cooking, the intake of fruits and vegetables, and in the frequency of cooking at home. - Food waste was reduced. Children ate more of the contents of their lunch boxes and they were willing to eat imperfect fruits and vegetables post-intervention.
Franceschi et al. 2021 [46]	- Daily frequency of vegetable and/or fruit consumption was significantly increased (from 36.6 to 51.7%; *p* < 0.001).
Teo et al. 2021 [39]	- At post-intervention, the EG increased their breakfast, lunch, dinner, and snack consumption frequency.
Nickel et al. 2021 [47]	- Urban children in EG improved dietary intake (4.6; 95% CI [0.9, 8.3]), and self-efficacy (5.3; 95% CI [1.0, 9.5]). - Boys who lived in higher-income neighborhoods in EG improved dietary intake.
Ben Cheikh et al. 2020 [48]	- Morning snack consumption decreased significantly in the EG (*p* = 0.009). - The proportion of children who had a morning snack habit decreased significantly between pre- and post-intervention (*p* = 0.000). - The consumption of cookies, cakes, and chocolates decreased significantly in the EG (*p* = 0.000).
Marshall et al. 2020 [34]	- There was a significant increase in FV intake and fiber intake in the EG. - There was a significant decrease in total fat intake and in the percentage of daily calories from sugar-sweetened beverages (*p* < 0.05). - Dietary data from parents showed significant increases in the combined fruit and vegetable intake (*p* < 0.05). - There were changes in the home nutrition environment which included: an increase in the frequency of cooking behaviors, in the use of nutrition facts labels, and in the availability of fruit and vegetable foods (*p* < 0.05).
Verdonschot et al. 2020 [49]	- There were no significant differences in (FV) consumption between EG1 and CG, and EG2 and CG. However, in EG2 there was an increase in FV consumption, and in EG1 and CG there was a decrease.
Siew et al. 2020 [40]	- In the attitude score, there were no significant changes for both groups over time.
Fisher et al. 2019 [35]	- In EG, there were significant differences between pre- and post-test in the knowledge of bedtime routines (*p* = 0.002). - Between groups, they only found significant differences in bedtime routines knowledge (*p* = 0.004).
Lin et al. 2019 [50]	-There were no significant differences in children’s healthy eating behaviors or active lifestyle behaviors between groups. - Children increased their interest in NASA space at the post-test, but this interest did not moderate the effect of Healthy Eating Active Living (HEAL) behaviors.
Qian et al. 2019 [41]	- The EG improved healthy eating behaviors at the post-test. There were significant differences between the pre- and post-test (*p* = 0.001). Moreover, the EG showed significantly higher scores than the CG (*p* = 0.025) in healthy eating behaviors. - In the EG there was a significantly larger portion of students who met the recommended level for several eating behaviors at the post-test. Here, there were differences between provinces. In Shandong, they met the recommended level for each of the nine eating behaviors (*p* < 0.001), and in Qinghay (lower family incomes), they met the recommended level for three of the nine behaviors (*p* < 0.05).
Kim et al. 2019 [51]	- There were significant changes in anthropometric variables in the EG (*p* < 0.05). - Total NQ score of the EG significantly increased (from 64.1 to 66.0, *p* < 0.05) - There was a higher fruit intake in EG than in CG (2.0 vs. 1.5, *p* < 0.05). - Moderation in the intake of hypercaloric foods was greater in the EG than in CG (0.5 vs. 1.2, *p* < 0.05). - Changes in meal regularity were significantly greater in EG than in CG (3.4 vs. 1.2, *p* < 0.05).
Brown et al. 2018 [52]	- There was a significant difference between the pre- and post-test in healthy snack attitude.
Zhou, 2018 [42]	- In EG there was a significant increase in nutritional attitude (*p* = 0.02) and nutritional behavior (*p* = 0.03) at post-test, but not in CG (*p* > 0.05).
Sharma et al. 2015 [37]	- There was a significant decrease in the amount of sugar consumed among children in EG compared to CG (*p* = 0.021).
Song et al. 2015 [38]	- Eating fruit for lunch is the unique factor that increased in the three groups with significant differences (*p* = 0.002 in CG, *p* = 0.006 in EG2, *p* = 0.011 in EG1). - EG1 significantly changed in eating vegetables for lunch (*p* = 0.007), eating any fruits the day before (*p* = 0.023), the number of days that they ate vegetables and fruits the previous week (*p* < 0.001), the self-efficacy to prepare FV at home (*p* = 0.034) and the perceptions of their peers’ fruit consumption (*p* = 0.025). Regarding the preference of students; berries and cherries had a significant increase only in CG (*p*=.006), while pears had a significant increase only in EG2 (*p* = 0.032). Oatmeal and granola had a significant change in EG2 (*p* = 0.036) and EG1 (*p* < 0.001), and grapes had a highly significant increase in the three groups (*p* < 0.001). Moreover, EG1 had significant differences in whole-grain noodles (*p* = 0.011), vegetables (*p* = 0.003), apples (*p* = 0.008), peaches and nectarines (*p* = 0.015), and squash, zucchini, and pumpkin (*p* = 0.032).

EG: Experimental Group; CG: Control Group; FV: Fruits and Vegetables; NASA: National Aeronautics and Space Administration; NQ: Nutritional Quotient.

## Data Availability

Not applicable.
